# LASSO regression-based machine learning model for differentiating spinal tuberculosis, pyogenic spondylitis, and endplate osteochondritis: development and clinical application

**DOI:** 10.3389/fcimb.2026.1834656

**Published:** 2026-06-02

**Authors:** Tuo Liang, Wenyang Chen, Yunfeng Nie, Zide Zhang, Xingming Lai, Kelin Li, Yonghui Wang, Yanjian Tang, Xubin Quan, Binhao Chen, Tongqing Luo

**Affiliations:** Department of Spinal Ward, Liuzhou People’s Hospital, Liuzhou, China

**Keywords:** clinical application, differential diagnosis, LASSO regression, machine learning, spinal tuberculosis

## Abstract

**Introduction:**

Accurate differentiation of spinal tuberculosis, pyogenic spondylitis, and endplate osteochondritis remains a clinical challenge due to overlapping clinical and radiological manifestations. This study aimed to construct and validate an interpretable machine learning model to assist the differential diagnosis of the three commonly confused spinal diseases.

**Methods:**

A total of 481 patients were retrospectively recruited from Liuzhou People’s Hospital between June 2020 and December 2024, including 247 cases of spinal tuberculosis, 92 cases of pyogenic spondylitis, and 142 cases of endplate osteochondritis. All enrolled participants were randomly divided into a training cohort and an internal validation cohort at a 7:3 ratio, with 338 and 143 patients respectively. Six machine learning algorithms were comprehensively evaluated, and the optimal LASSO regression model was constructed based on 12 screened core features. SHAP analysis was employed to interpret global feature importance and individual predictive contributions. Additionally, an open-access web-based prediction calculator was developed to support clinical decision-making.

**Results:**

In the validation cohort, the LASSO model achieved AUC values of 0.838 for spinal tuberculosis, 0.683 for pyogenic spondylitis, and 0.897 for endplate osteochondritis. SHAP analysis quantified the predictive contribution of each feature and enhanced model interpretability. A user-friendly online prediction tool was successfully constructed for clinical auxiliary use.

**Conclusion:**

This study established an interpretable machine learning model and a supporting web calculator. The model exhibits good diagnostic efficiency for spinal tuberculosis and endplate osteochondritis and provides a practical auxiliary tool for clinical differential diagnosis of the three spinal conditions.

## 1 Introduction

Spinal tuberculosis (STB), also known as Pott’s disease, is the most prevalent form of musculoskeletal tuberculosis, accounting for approximately 50% of all osteoarticular tuberculosis cases (Kumar et al., 2022). It represents a significant global health challenge, particularly in developing regions and remote areas with limited healthcare resources. The insidious onset and non-specific clinical manifestations of STB—often presenting as back pain, constitutional symptoms, and potential neurological deficits—frequently lead to diagnostic delays, resulting in severe complications such as kyphotic deformities and spinal cord injury. Traditional diagnostic methods for STB include magnetic resonance imaging (MRI), computed tomography (CT), interferon-gamma release assays (IGRAs), and microbiological or histopathological confirmation (Xu et al., 2025; Nduba et al., 2024; Yan et al., 2023). While MRI remains the imaging modality of choice due to its high sensitivity in detecting early marrow edema, disc destruction, and paravertebral abscesses, its findings often overlap with those of other spinal pathologies, making differentiation challenging (De Aguiar Martins et al., 2025; Xu et al., 2025). Moreover, conventional laboratory tests like erythrocyte sedimentation rate (ESR) and C-reactive protein (CRP) are often elevated but lack specificity. The gold standard for diagnosis—mycobacterial culture or biopsy—is invasive, time-consuming, and not always readily available, highlighting an urgent need for rapid, accurate, and non-invasive diagnostic tools (Ştefanescu et al., 2021).

In recent years, artificial intelligence (AI) and machine learning (ML) have emerged as powerful adjuncts in medical imaging and clinical diagnostics, offering potential solutions to these challenges. Deep learning models, particularly convolutional neural networks (CNNs), have been developed for computer-aided diagnosis (CAD) of STB from CT images (Green et al., 2022; Sarawagi et al., 2024). For instance, a multimodal feature fusion CNN that integrates handcrafted image features with self-extracted deep learning features has demonstrated high accuracy in classifying spinal TB cases, with models like VGG-11 achieving optimal performance (Li et al., 2022b). Similarly, advanced architectures such as Mask R-CNN, enhanced with attention mechanisms, have been employed for the precise segmentation of STB lesions. Beyond imaging analysis, ML algorithms are increasingly applied to routine laboratory parameters to create diagnostic and predictive models (Verrou et al., 2025). Multiple studies have leveraged ML algorithms—including logistic regression, random forest, and support vector machines—to identify key hematological and inflammatory biomarkers that differentiate STB from other spinal infections (Ahuja et al., 2022). In line with this, a nomogram developed via least absolute shrinkage and selection operator (LASSO) regression and ROC analysis with the monocyte-to-lymphocyte ratio (MLR) and other blood-based inflammatory markers achieved AUC values of 0.801 in the training set and 0.861 in the external validation set for diagnosing STB and revealed an independent association between MLR and disease severity (Chen et al., 2022). Extending these efforts, ML-based predictive models such as random forest algorithms have been developed to forecast complications like spinal cord injury in STB patients, with monocyte count identified as a significant predictor (Cursi et al., 2023).

A paramount challenge in STB management is its differentiation from other spinal conditions that present with similar clinical and radiological features, primarily pyogenic spondylitis (PS), brucellar spondylitis, and, in some instances, non-infectious inflammatory conditions like endplate osteochondritis. PS, often caused by Staphylococcus aureus, typically presents more acutely with higher inflammatory markers, while brucellar spondylitis is endemic in certain regions and shares several overlapping features with STB. In atypical cases, distinction based solely on clinical presentation or conventional imaging can be difficult. Several ML models have shown efficacy in addressing this issue, but almost all have been designed for binary classification tasks—for instance, differentiating STB from PS (Tien et al., 2025) or STB from brucellar spondylitis (Ahuja et al., 2022). While these models achieve respectable performance (AUC 0.70–0.90), they do not reflect the real-world clinical scenario in which clinicians must simultaneously discriminate among multiple competing diagnoses. Furthermore, the range of ML algorithms explored in spinal infection differential diagnosis has remained narrow, largely limited to logistic regression, random forest, and support vector machines. To the best of our knowledge, other powerful and widely used algorithms—such as LASSO regression, K-nearest neighbors (KNN), extreme gradient boosting (XGBoost), decision trees (DT), and ridge regression—have rarely, if ever, been systematically evaluated in this domain (Xia et al., 2025; Chen et al., 2025; Gu et al., 2025). The potential of these algorithms to capture complex, nonlinear interactions among routine clinical variables for multi-class discrimination of spinal infections remains unexplored, representing a critical knowledge gap.

To address these limitations, the present study introduces a comprehensive ML framework for the simultaneous, multi-class differential diagnosis of the three most common and clinically confounding spinal diseases: STB, PS, and endplate osteochondritis. For the first time in this context, we systematically evaluate and compare a diverse panel of ML algorithms—including LASSO regression, KNN, random forest (RF), XGBoost, DT, and ridge regression—using exclusively the most widely available and easily obtainable routine clinical and laboratory parameters. These features were deliberately selected to maximize generalizability and facilitate adoption in resource-limited settings where advanced imaging or specialized tests may be unavailable. Importantly, we further translate our optimal model into a freely accessible, user-friendly web-based tool, effectively bridging the gap between algorithm development and practical clinical decision support. By moving beyond binary classification and leveraging an expanded suite of ML techniques, this study aims to provide a more realistic, accurate, and clinically actionable aid for the early differentiation of spinal diseases, with the ultimate goal of reducing diagnostic delays and improving patient outcomes, especially in high-burden, resource-constrained regions.

## 2 Materials and methods

### 2.1 Patients

We conducted a retrospective cohort study using clinical data from over 10,000 patients hospitalized at Liuzhou People’s Hospital between June 2020 and December 2024. Data were extracted from the hospital’s electronic medical record system (Zhiye Record System, version 2.3.137). The study focused on three distinct spinal pathologies: STB, PS, and endplate osteochondritis (Modic changes, MC).

To ensure diagnostic homogeneity, the gold standard for case inclusion was predefined for all three groups, and the most rigorous diagnostic logic was applied consistently. Histopathological and/or microbiological examination was adopted as the gold standard for all three conditions:

- STB: The gold standard was detection of Mycobacterium tuberculosis in lesion tissue or pus (positive acid-fast stain, positive mycobacterial culture, or positive Xpert MTB/RIF assay) or the demonstration of typical tuberculous granulomas with caseous necrosis and Langhans giant cells.
- PS: The gold standard was a positive bacterial culture from blood or lesion tissue or histopathological evidence of pyogenic inflammation (neutrophilic infiltration, abscess formation).
- Endplate osteochondritis: Given that MC represent non-infectious degeneration for which routine open biopsy is clinically unwarranted, the gold standard was defined as the exclusion of infection (i.e., negative microbiological studies and the absence of infectious pathology), combined with characteristic imaging features and a non-progressive clinical course over at least 12 months of follow-up. This definition is consistent with clinical guidelines and ethical standards.

In a subset of suspected cases where pathological or microbiological evidence was unavailable, a predefined set of clinical diagnostic criteria was adopted as a supplementary basis for inclusion. These criteria served as operational definitions in specific circumstances rather than constituting the gold standard itself. Symmetric and rigorous inclusion logic was maintained across the three groups, as detailed in **Supplementary Table 1**.

### 2.2 Statistical analysis of baseline characteristics

First, the Shapiro-Wilk test was used to assess the normality of the 50 included variables. For normally distributed variables, overall comparisons among groups were performed with Welch’s one-way analysis of variance (ANOVA), and pairwise comparisons were conducted using the Games-Howell test. For non-normally distributed variables, overall comparisons were performed with the Kruskal-Wallis H test, and pairwise comparisons were conducted using Dunn’s test with Bonferroni correction. Data processing and analysis were conducted using R software (version 4.5.1), in strict adherence to standard medical statistical principles to ensure scientific rigor and result reliability.

### 2.3 Predictive model construction and evaluation

For predictive modeling, we constructed and evaluated models based on six machine learning algorithms: LASSO regression, KNN, RF, XGBoost, DT, and ridge regression. To robustly identify the optimal algorithm, a total of 1000 bootstrap resampling iterations were performed. In each iteration, the dataset was randomly stratified into a training set and an internal validation set at a 7:3 ratio to preserve class distribution and ensure generalizability; all six models were trained and validated, and the best-performing model was selected according to the AUC and accuracy, with sensitivity, specificity, and the F1−score serving as supplementary metrics. The algorithm selected most frequently across the 1000 iterations was defined as the optimal model, and LASSO regression emerged as the most frequently selected algorithm. Subsequently, an additional 1000 LASSO modeling runs were conducted to evaluate whether performance metrics differed between the model built with λmin and that built with λ1se (the one−standard−error rule) (Friedman et al., 2010).

### 2.4 Stable feature selection via repeated LASSO

An initial set of 50 clinical variables was collected. To minimize overfitting and the bias introduced by a single random data partition, stable feature selection was conducted via 1000 iterations of LASSO regression (with the penalty parameter λmin) under bootstrap resampling. Across the 1000 iterations, the number of variables retained by the λmin criterion ranged from 13 to 50, with a mean of 25. This provided an objective characterization of the stable selection range and effectively avoided the selection bias that can arise from a single randomized dataset. Based on the stable selection range and the lower bound of 13 variables, we prospectively designed three tiered feature subsets consisting of the top 10, 12, and 15 variables most frequently selected across the 1000 LASSO iterations. Models were constructed with each subset and their performance was compared. This tiered design allowed us to balance model parsimony and generalizability while objectively evaluating the impact of feature dimensionality on predictive performance.

### 2.5 Model calibration, interpretation, and clinical deployment

The calibration of the final model was assessed using calibration curves, and its clinical net benefit was evaluated through decision curve analysis (DCA). To elucidate the model’s decision−making process, SHapley Additive exPlanations (SHAP) values were employed. SHAP summary plots, bar plots, dependence plots, and individual force plots were generated to visualize the global importance of each predictor and its contribution to individual predictions (Lundberg et al., 2019). Ultimately, the model was deployed as a web−based clinical application tool to facilitate practical use in clinical settings.

### 2.6 Ethics approval and informed consent

This study was ethically approved by the Ethics Committee of the Liuzhou People’s Hospital and registered in the Chinese Clinical Trial Registry. The study was in compliance with the Helsinki Declaration. All patients signed written informed consent forms prior to enrollment.

## 3 Results

### 3.1 Baseline characteristics of patients

**Figure 1:**
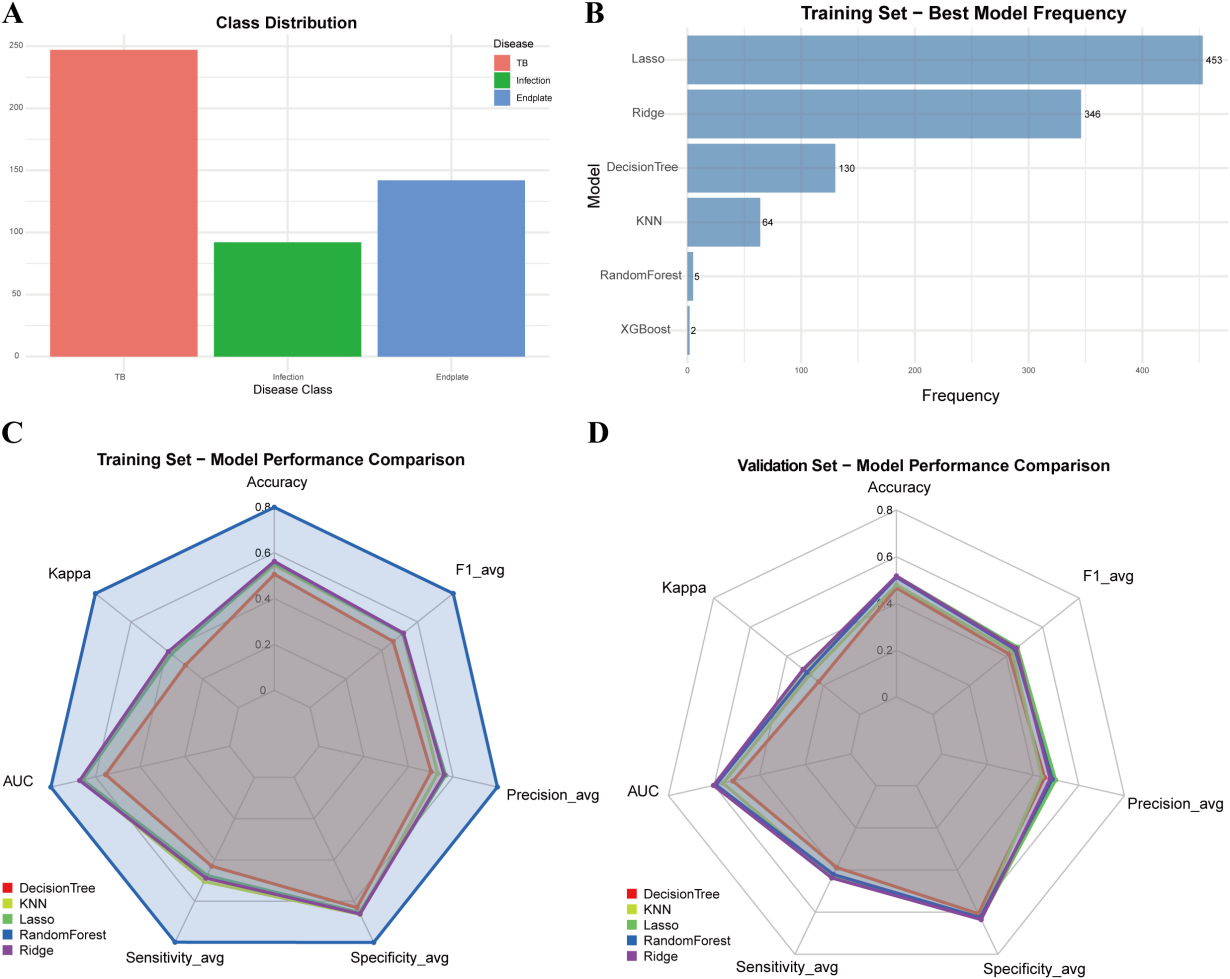
**(A)** Disease class distribution of 481 patients. **(B)** Frequency statistics of the optimal ML model for 1000 hold out test sets. Radar chart comparison of six machine learning models’ performance metrics in **(C)** training and **(D)** validation cohorts.

From June 2020 to December 2024, a total of 481 patients with complete clinical and laboratory profiles, diagnosed with STB, PS, or endplate osteochondritis, were included in the final analysis. The cohort comprised 247 patients with STB, 92 with PS, and 142 with endplate osteochondritis (**Figure 1A**). Intergroup comparisons of all baseline indicators and their corresponding statistical differences are detailed in **Supplementary Table 2**.

### 3.2 Performance evaluation of multiple ML models

All 481 enrolled patients were randomly divided into a training cohort and an internal validation cohort at a ratio of 7:3. We developed six machine learning models, including least absolute shrinkage and selection operator LASSO regression, KNN, RF, XGBoost, DT, and ridge regression. Initial evaluation via repeated iterations indicated that the LASSO regression model was most frequently selected as the optimal model (**Figure 1B**). Radar charts were used to comprehensively compare the classification performance of all six models across multiple metrics—including AUC, accuracy, sensitivity, specificity, precision, and F1-score—calculated from confusion matrices of both the training (n = 338) and validation cohorts (n = 143) (**Figures 1C**, **D**).

**Figure 2:**
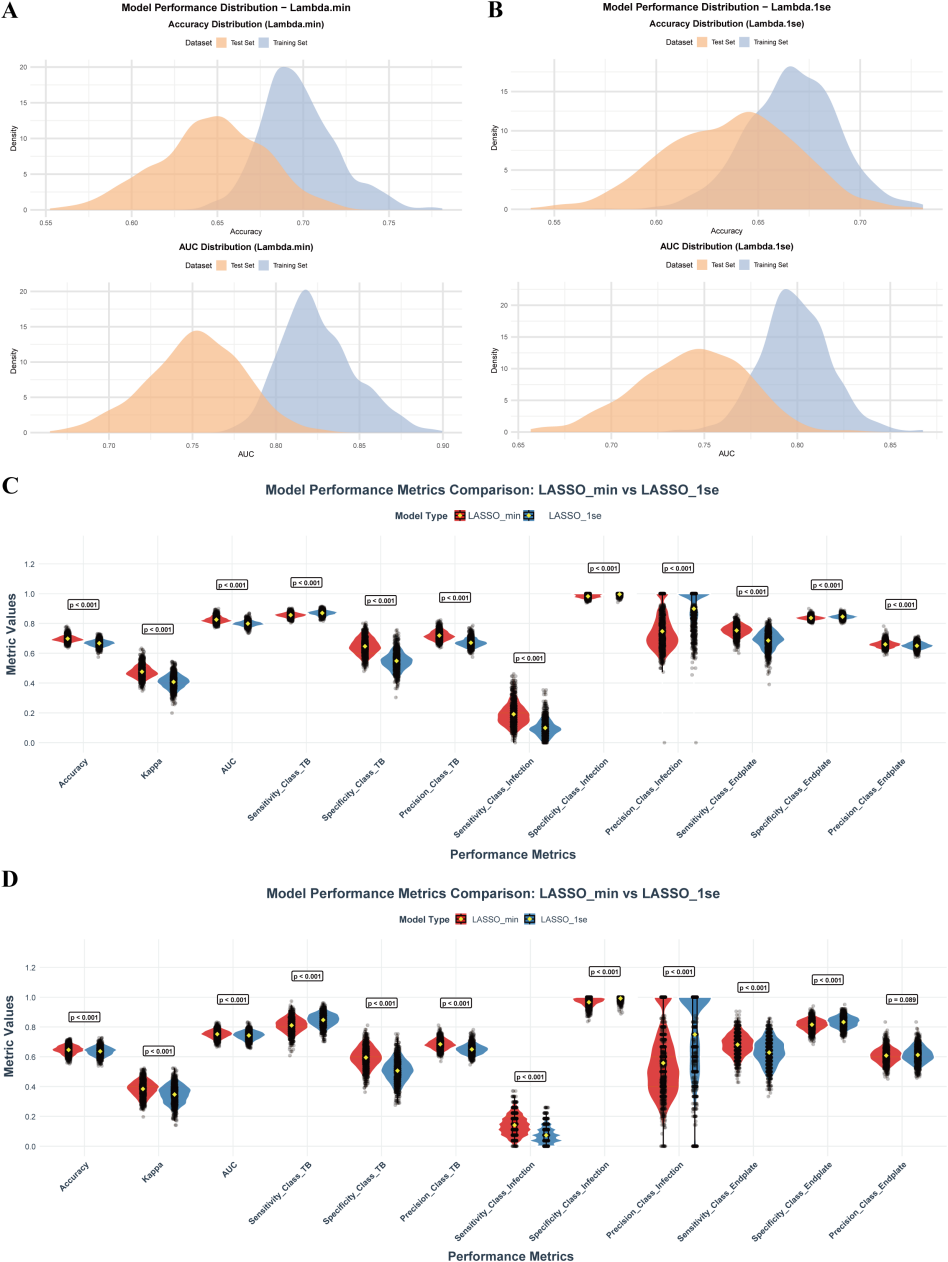
**(A)** Frequency distribution of accuracy and AUC for the training set across 1000 LASSO regression simulations. **(B)** Frequency distribution of accuracy and AUC for the validation set across 1000 LASSO regression simulations. **(C)** Comparison of all evaluation statistic metrics for LASSO regression in the training set using the min λ and 1se λ (1000 hold out test sets). **(D)** Comparison of all evaluation statistic metrics for LASSO regression in the validation set using the min λ and 1se λ (1000 hold out test sets).

Subsequent analysis of 1000 repeated modeling runs for the LASSO regression model revealed that the model built using the λmin penalty parameter consistently outperformed the λ1se-based model in terms of accuracy and AUC across both training and validation sets (**Figures 2A**, **B**). This performance advantage of the λmin-based model was consistent across all other evaluated metrics (**Figures 2C**, **D**), so λmin was selected for final model construction.

**Figure 3:**
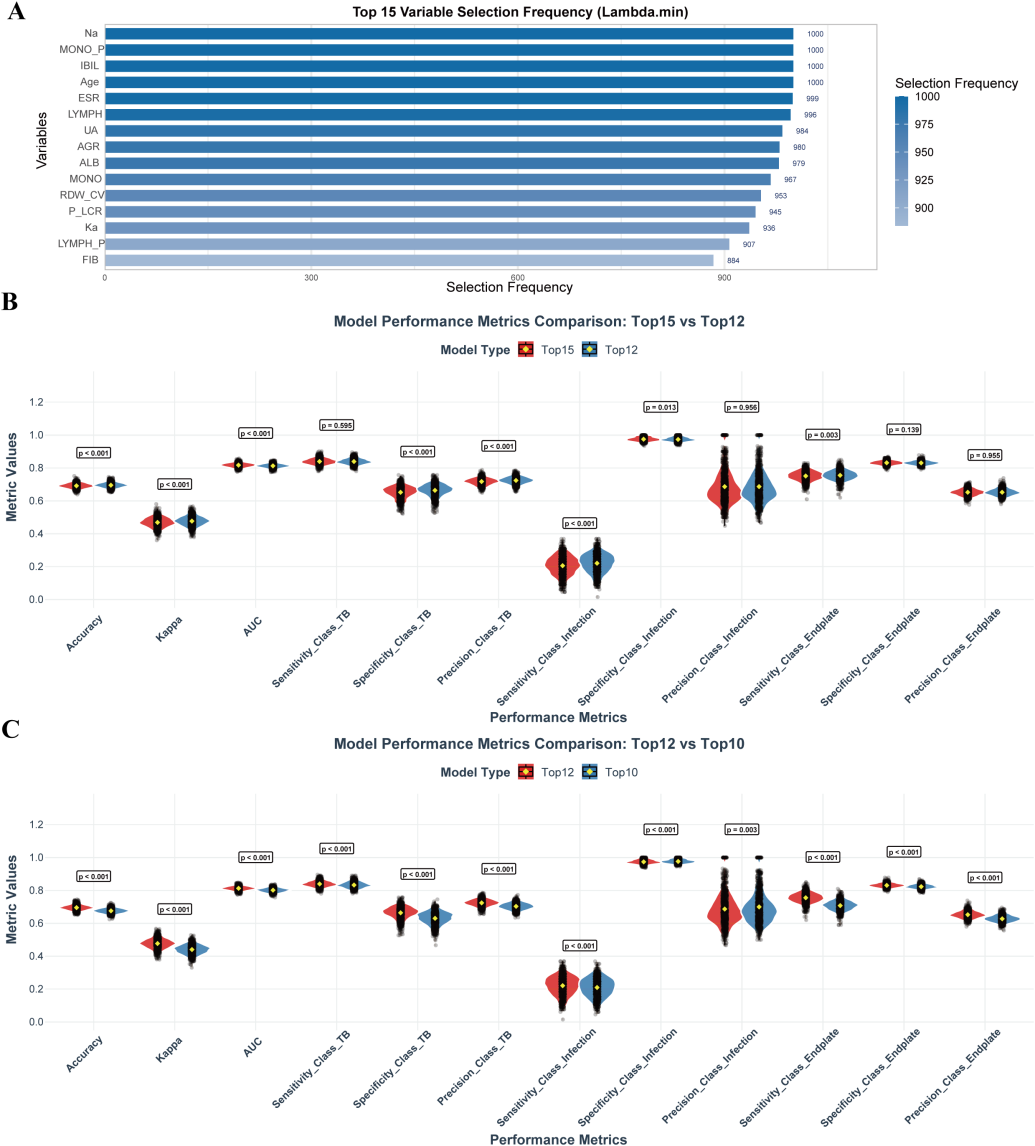
**(A)** Frequency statistics of variable selection across 1,000 LASSO regression runs using the min λ. **(B)** Comparison of model parameters from 1,000 bootstrap replications using the top 15 versus the top 12 most frequently selected variables (based on the min λ). **(C)** Comparison of model parameters from 1,000 bootstrap replications using the top 12 versus the top 10 most frequently selected variables (based on the min λ).

To improve model parsimony and clinical applicability without compromising predictive performance, we refined the feature set by sequentially retaining the top 15, top 12, and top 10 most frequently selected features (**Figure 3A**) and compared model performance across the three feature subsets. **Figure 3B** compares the performance of the Top15 and Top12 feature-based models across 12 evaluation metrics, showing that the Top12 model outperformed the Top15 model in 8 metrics (Accuracy, Kappa, AUC, specificity for TB, precision for TB, sensitivity for infection, sensitivity for endplate, specificity for infection and specificity for endplate) with no significant differences in the remaining 4 metrics. **Figure 3C** further compares the Top12 and Top10 feature models, demonstrating that the Top12 model achieved superior performance in 10 metrics and was non-inferior in the rest. Thus, the model built with the top 12 features was selected as the optimal balance of performance and parsimony.

**Figure 4:**
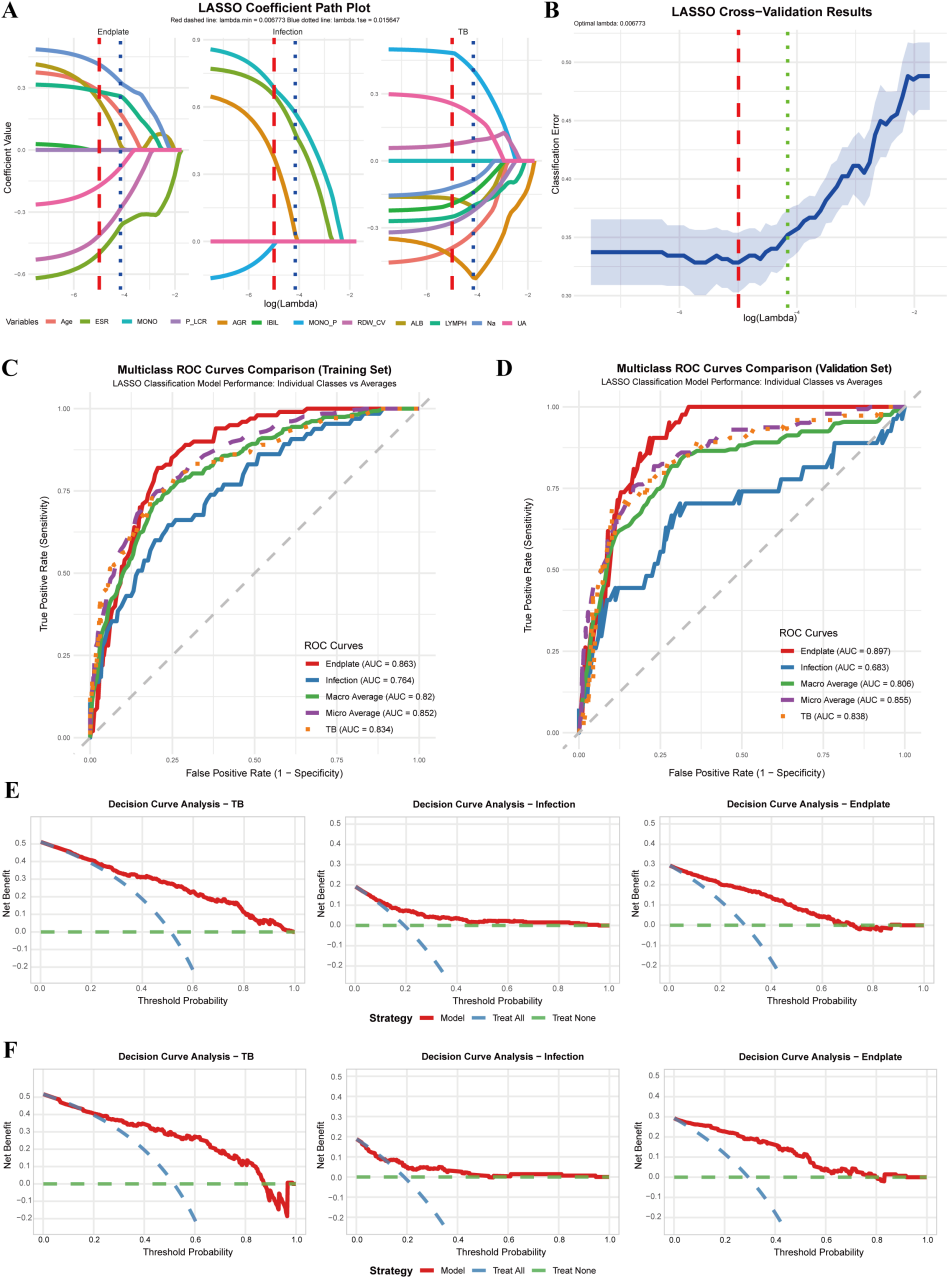
**(A)** The LASSO coefficient trajectories. **(B)** A curve of cross-validation error representing the selection of the tuning parameter (λ). ROC curves of the LASSO model for predicting different disease class in the training **(C)** and validation set **(D)**. Comparison of decision curve analysis of the LASSO model in the training **(E)** and validation set **(F)**.

Based on the above analyses, LASSO regression was chosen as the core algorithm for the final predictive model. Feature refinement was performed using the optimal λmin penalty parameter, balancing model simplicity and predictive performance (**Figures 4A**, **4B**). ROC curve analysis demonstrated that in the training cohort, the LASSO regression model achieved AUCs of 0.834, 0.764, and 0.863 for discriminating STB, PS, and endplate osteochondritis, respectively (**Figure 4C**). Corresponding AUCs in the validation cohort were 0.838, 0.683, and 0.897, confirming robust discriminative ability (**Figure 4D**). We have also listed all relevant parameters of the final model in **Table 1**.

**Table 1:** The performance of the LASSO regression model.

	Training set		Validation set	
Metric	Estimate	95% CI	Estimate	95% CI
Accuracy	0.6834	[0.6309, 0.7327]	0.7063	[0.6244, 0.7794]
Kappa	0.4594	[0.4098, 0.5090]	0.4921	[0.4174, 0.5667]
AUC_Macro	0.8202	[0.7828, 0.8580]	0.8059	[0.7375, 0.8708]
Sensitivity_TB	0.7186	[0.6506, 0.7799]	0.7241	[0.6179, 0.8146]
Specificity_TB	0.7842	[0.7065, 0.8494]	0.8036	[0.6757, 0.8977]
Precision_TB	0.8266	[0.7618, 0.8798]	0.8514	[0.7496, 0.9234]
NPV_TB	0.6606	[0.5829, 0.7324]	0.6522	[0.5279, 0.7629]
AUC_TB	0.8337	[0.7911, 0.8764]	0.8376	[0.7693, 0.9060]
Sensitivity_Infection	0.6071	[0.4058, 0.7850]	0.6364	[0.3079, 0.8907]
Specificity_Infection	0.8452	[0.8000, 0.8836]	0.8485	[0.7757, 0.9049]
Precision_Infection	0.2615	[0.1603, 0.3854]	0.2593	[0.1111, 0.4628]
NPV_Infection	0.9597	[0.9290, 0.9797]	0.9655	[0.9141, 0.9905]
AUC_Infection	0.7638	[0.6991, 0.8285]	0.6833	[0.5522, 0.8143]
Sensitivity_Endplate	0.6396	[0.5430, 0.7286]	0.6889	[0.5335, 0.8183]
Specificity_Endplate	0.8722	[0.8217, 0.9127]	0.8878	[0.8080, 0.9426]
Precision_Endplate	0.7100	[0.6107, 0.7964]	0.7381	[0.5796, 0.8614]
NPV_Endplate	0.8319	[0.7783, 0.8771]	0.8614	[0.7784, 0.9221]
AUC_Endplate	0.8630	[0.8241, 0.9019]	0.8967	[0.8472, 0.9463]
Macro_Sensitivity	0.6551	[0.5487, 0.6543]	0.6831	[0.5405, 0.6959]
Macro_Specificity	0.8339	[0.7901, 0.8444]	0.8466	[0.7829, 0.8683]
Macro_Precision	0.5994	[0.5830, 0.7290]	0.6162	[0.5657, 0.7970]
Macro_NPV	0.8174	[0.8044, 0.8626]	0.8264	[0.8007, 0.8891]

DCA further evaluated the clinical utility of the model. As shown in **Figures 4E**, **F**, the LASSO regression model provided a significantly higher net clinical benefit than the “treat all” and “treat none” strategies across the threshold probability range of 0.0 to 0.8 for all three disease categories, particularly for STB and endplate osteochondritis.

**Figure 5:**
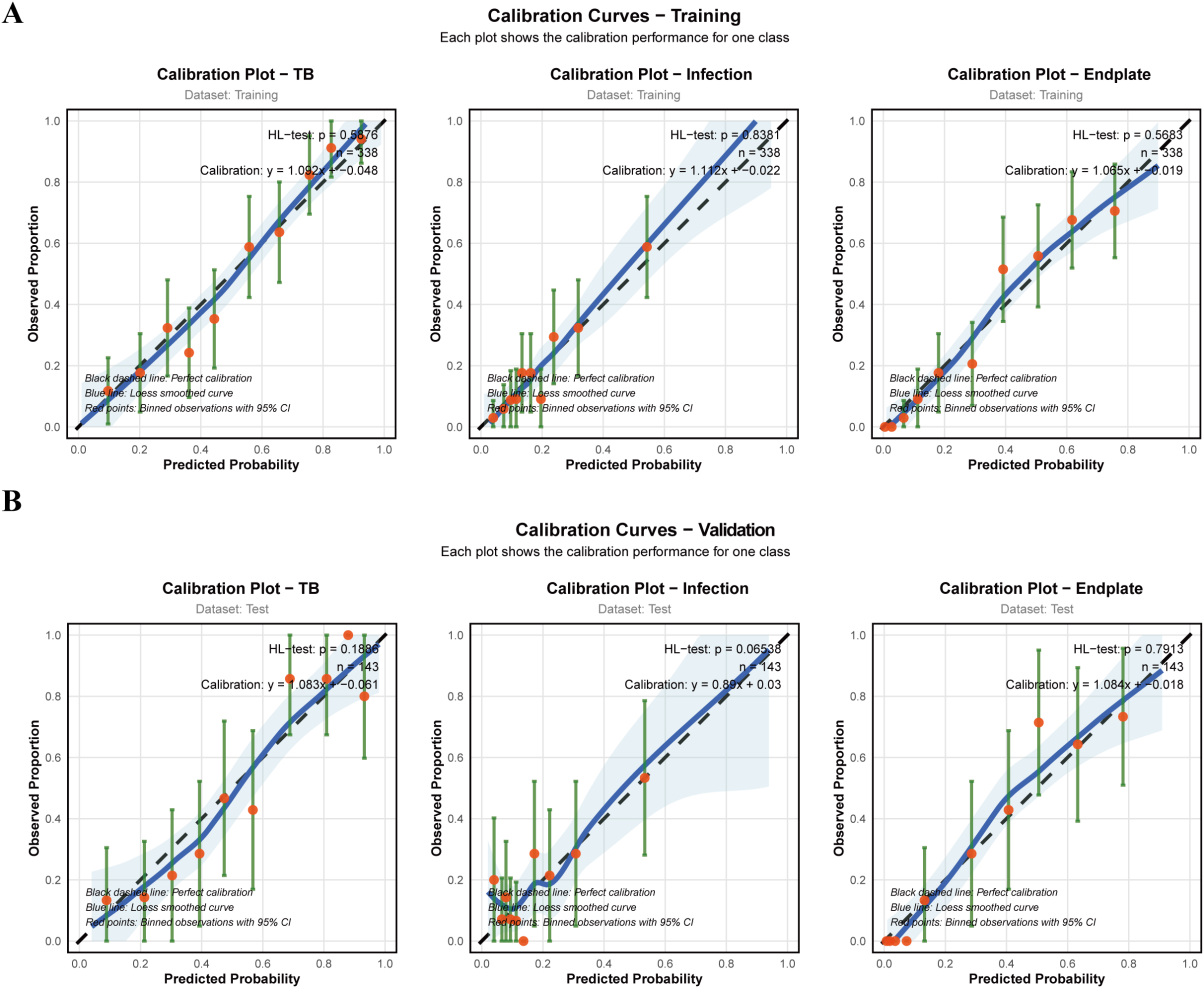
Calibration curves for predicting disease class in the training cohort **(A)** and validation cohort **(B)**.

Calibration curves were plotted to assess the agreement between predicted probabilities and observed outcomes. In the training cohort (**Figure 5A**), the Hosmer-Lemeshow test yielded P-values of 0.59, 0.84, and 0.56 for STB, PS, and endplate osteochondritis, respectively; corresponding P-values in the validation cohort (**Figure 5B**) were 0.19, 0.07, and 0.79. All P-values were greater than 0.05, indicating good calibration with no significant deviation between predicted and observed probabilities. The slopes and intercepts of the calibration curves further confirmed the absence of systematic overestimation or underestimation bias in model predictions.

### 3.3 SHAP-Based interpretability for LASSO regression

**Figure 6:**
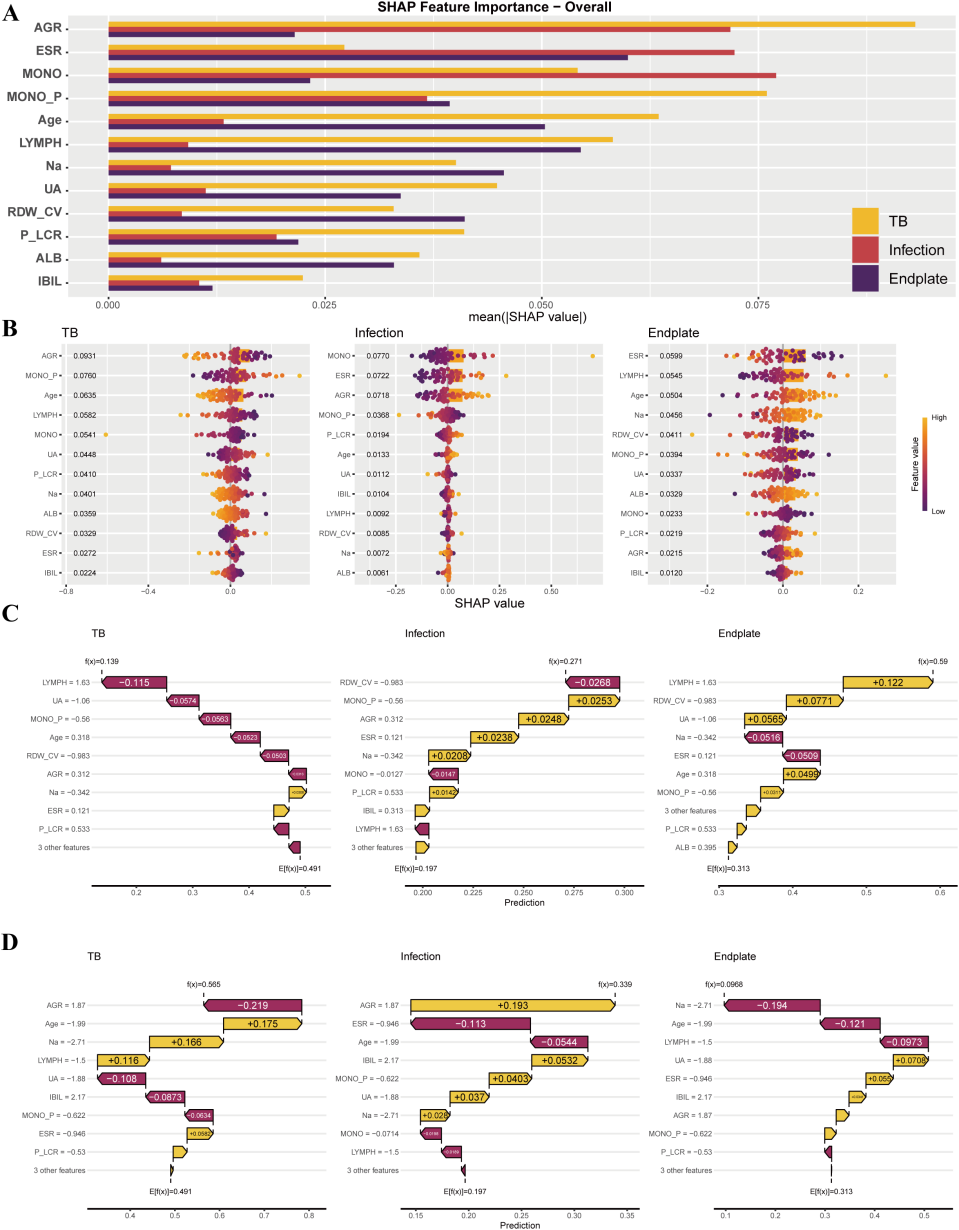
SHAP-based interpretation of the LASSO regression model. **(A)** SHAP bar plot ranking the top predictive features by their mean absolute SHAP value, indicating global feature importance. **(B)** SHAP summary plot illustrating the direction and magnitude of each feature’s effect on the model’s output across the dataset. **(C)** SHAP force plot for an individual patient correctly predicted as endplate osteochondritis. **(D)** SHAP force plot for an individual patient correctly predicted as TB.

For global interpretability, the SHAP bar plot (**Figure 6A**) ranks the 12 included features according to their mean absolute SHAP values, reflecting their overall importance to model predictions. The top contributing features were albumin/globulin ratio (AGR), ESR, and monocyte absolute count (MONO), with mean absolute SHAP values of 0.0621, 0.0531, and 0.0515, respectively. Notably, the contribution of individual features varied significantly among different disease categories, highlighting their distinct roles in differentiating between the three spinal conditions. The SHAP summary plot (**Figure 6B**) further illustrates the direction and magnitude of each feature’s influence on predictions: a positive SHAP value indicates that higher feature values increase the predicted probability of the target disease category, while a negative value indicates the opposite.

For local interpretability, we visualized individual prediction cases to demonstrate the model’s decision logic. In the case presented in **Figure 6C**, the model calculated the expected baseline prediction value E[f(x)] as 0.491 for STB, 0.197 for PS, and 0.313 for endplate osteochondritis. The sum of SHAP contributions from the 12 features shifted the prediction probabilities to 0.139 for STB, 0.271 for PS, and 0.590 for endplate osteochondritis, leading to the final classification of this sample as endplate osteochondritis. Conversely, for the sample in **Figure 6D**, the SHAP contributions shifted the prediction probabilities to 0.565, 0.339, and 0.097, respectively, resulting in a classification of STB. These local SHAP visualizations provide clear, case-specific explanations of how each clinical indicator influences the model’s output, enhancing the transparency and clinical trustworthiness of the predictive tool.

### 3.4 Development of an online interactive clinical decision support tool

**Figure 7:**
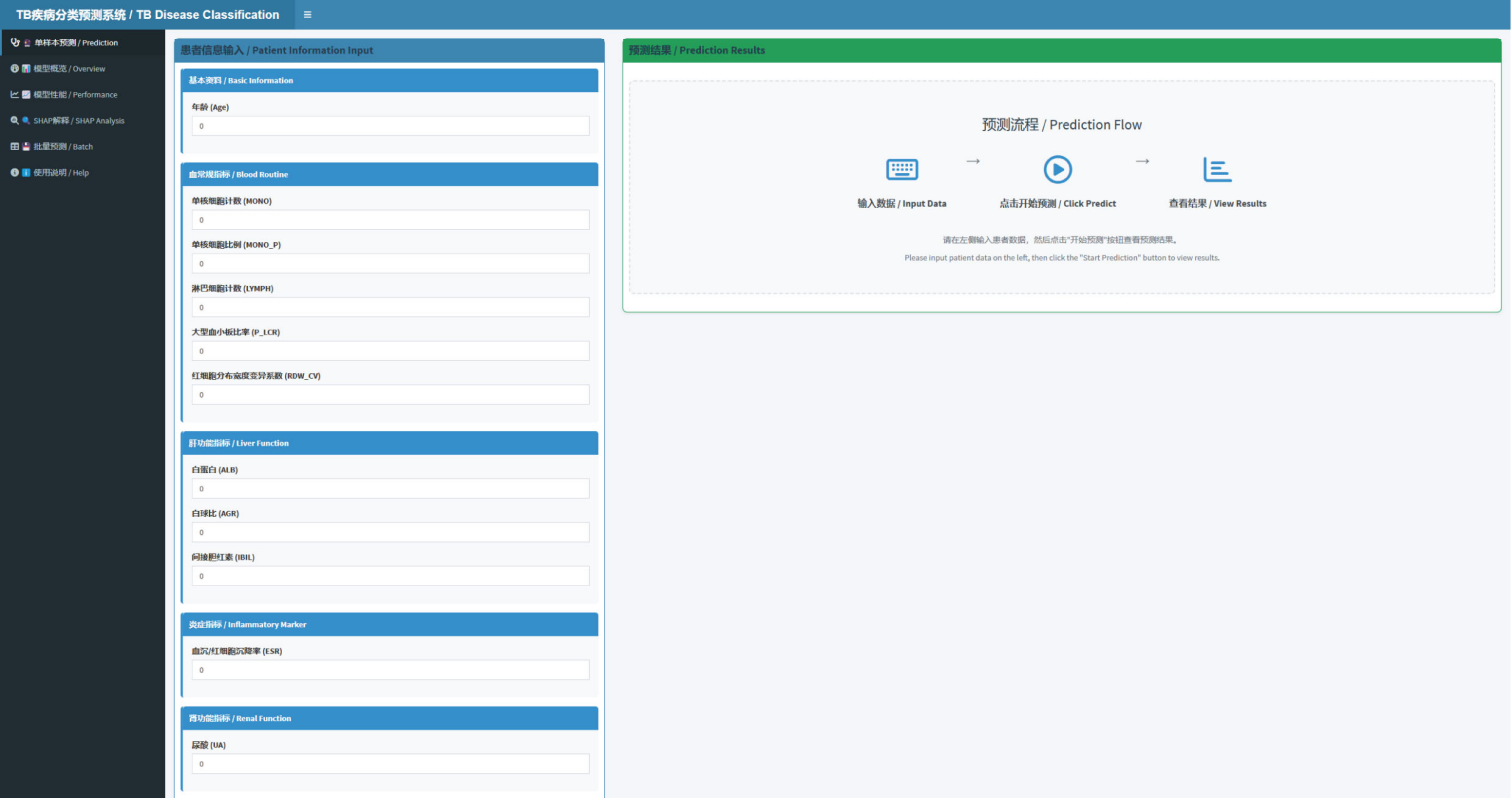
An efficient tool for distinguishing among three common and easily confused spinal disorders.

**Figure 8:**
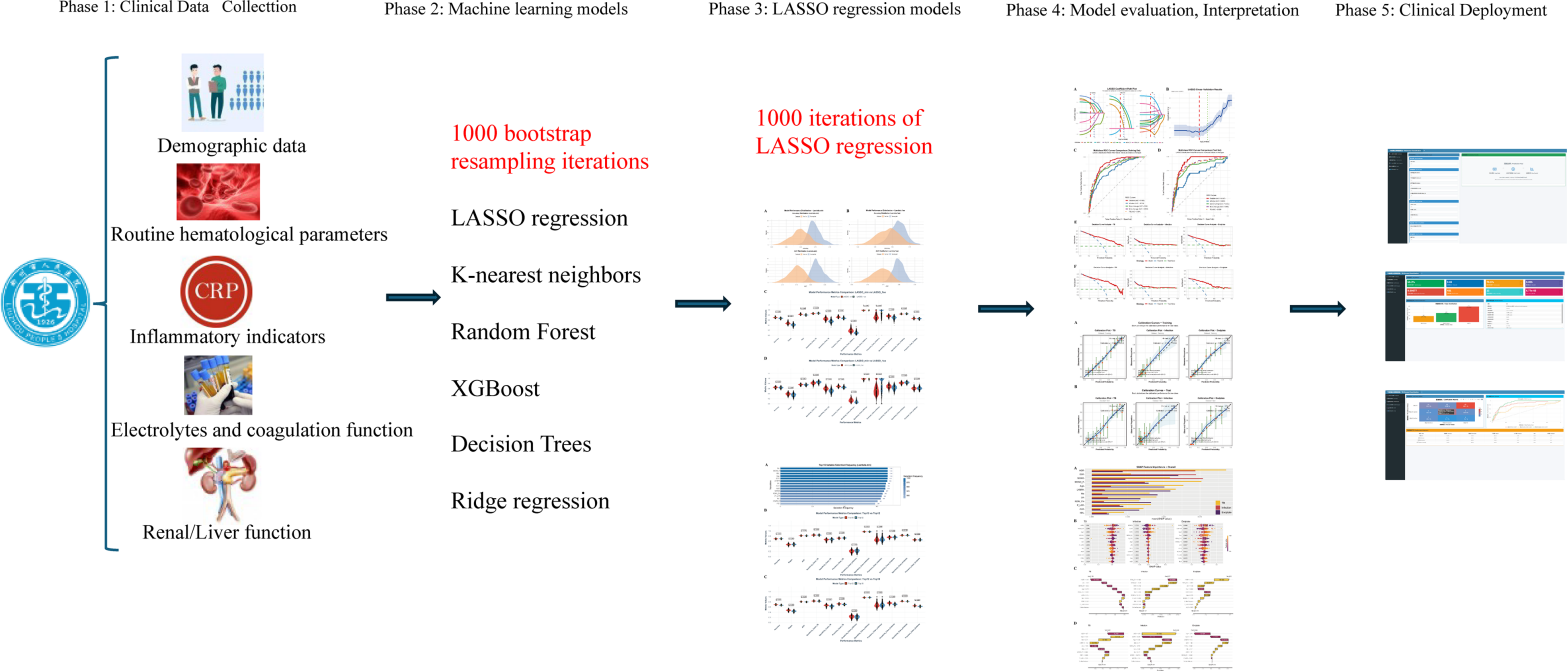
Complete research flowchart.

To facilitate the clinical translation and practical implementation of our predictive model, we deployed the final LASSO regression model—constructed using 12 routine clinical indicators (serum sodium, monocyte ratio [MONO_P], monocyte absolute count [MONO], indirect bilirubin [IBIL], age, ESR, lymphocyte absolute count [LYMPH], uric acid [UA], albumin [ALB], albumin/globulin ratio [AGR], red cell distribution width [RDW-CV], and platelet crit [PLCR])—as a freely accessible, user-friendly online prediction calculator (**Figure 7**). This interactive tool enables clinicians to input routine patient parameters and generate personalized, real-time risk predictions for the three common spinal disorders, thereby providing objective auxiliary support for clinical decision-making. The online calculator is publicly available at: https://liangtuo1234.shinyapps.io/Spine_Disease_Prediction/. The Complete research flowchart is presented in **Figure 8**.

## 4 Discussion

Accurate differentiation of STB, PS, and endplate osteochondritis remains a critical clinical diagnostic challenge, due to highly overlapping clinical manifestations and imaging characteristics among the three conditions. To address this unmet clinical need, the present study constructed and validated a ternary classification machine learning model for differential diagnosis of the three spinal disorders. Unlike most previous studies that only focused on binary classification between two spinal diseases, this work attempted simultaneous three-way differentiation based on routine hematological and inflammatory indicators. Nevertheless, considering the limitations of the single-center retrospective design, inter-group sample imbalance, and the moderate diagnostic efficacy of the PS subgroup, the innovation and clinical value of the model should be interpreted cautiously rather than overgeneralized.

In terms of model diagnostic performance, the LASSO regression model yielded acceptable discriminative efficacy in the training cohort, with AUC values of 0.834, 0.764, and 0.863 for STB, PS, and endplate osteochondritis, respectively. In the internal validation cohort, the corresponding AUCs were 0.838, 0.683, and 0.897. Compared with previous ML predictive models for spinal infectious diseases, which mostly reported AUCs ranging from 0.83 to 0.86 for binary differentiation (Xia et al., 2025; Tien et al., 2025), our model achieved favorable predictive performance for STB and endplate osteochondritis. However, the model showed only moderate discriminative ability for the PS subgroup, with the validation AUC decreasing to 0.683, which was significantly inferior to the other two categories. Further DCA analysis demonstrated that the model maintained a positive net clinical benefit across the threshold range of 0.0–0.8, indicating certain clinical decision-making value; calibration analysis also revealed good consistency between predicted probability and actual observation in both training and validation cohorts without obvious systematic deviation. Overall, the model exhibited stable performance in identifying STB and endplate osteochondritis, while the diagnostic ability for PS was relatively limited.

One prominent limitation of this study is the imbalanced sample size among the three groups: 247 cases of STB, 92 cases of PS, and 142 cases of endplate osteochondritis. The PS group had the smallest sample volume, which is an inevitable reality in clinical retrospective enrollment but also an important factor restricting model performance. An imbalanced sample distribution tends to make the model biased toward categories with larger sample sizes during algorithm training, thereby reducing feature learning efficiency and generalization ability for small-sample subgroups. This also reasonably explains why the PS subgroup obtained the lowest AUC in both training and validation sets, with only moderate diagnostic accuracy. In view of this, we do not overstate the universal stability and broad applicability of the model; instead, we acknowledge that the predictive performance for PS needs to be further improved by expanding the sample size in subsequent studies.

The model finally screened 12 core routine clinical and laboratory features, including serum sodium, monocyte ratio and absolute count, indirect bilirubin, age, ESR, lymphocyte absolute count, uric acid, albumin, AGR, RDW-CV, and PLCR. These indicators cover inflammation, nutrition, metabolism, and hematological cellular parameters, providing a reasonable pathophysiological basis for model classification logic.

Monocyte proportion, monocyte absolute count, and ESR are classic systemic inflammatory indicators (Chen et al., 2022; Na et al., 2023; Nduba et al., 2024; Rajamanickam et al., 2021). In our cohort, these inflammatory indicators presented distinct expression patterns among the three groups, and SHAP analysis further confirmed their high global feature importance. As infectious diseases, STB and PS can induce more intense systemic inflammatory activation, whereas endplate osteochondritis is dominated by localized degenerative changes with milder inflammatory response, which becomes an important basis for model differentiation.

Albumin, AGR, and uric acid are representative nutritional and metabolic indicators (Nduba et al., 2024). AGR are recognized indicators reflecting nutritional status and chronic disease burden (Perumal et al., 2024). Consistent with existing literature, patients with chronic STB tended to have lower albumin and AGR in our real-world cohort, which helped distinguish STB from PS and degenerative endplate lesions. Uric acid, which is influenced by both inflammatory response and metabolic level, also showed differential predictive contributions in SHAP analysis, supporting its auxiliary value in differentiating the three conditions (Hamed et al., 2021; Li et al., 2022a).

Lymphocyte absolute count, RDW-CV, and PLCR are important hematological cellular indices reflecting bone marrow hematopoietic response and systemic stress status. Chronic inflammation and malnutrition in STB patients are often accompanied by abnormal lymphocyte count and elevated RDW-CV (Tomic et al., 2024). while PLCR is closely related to inflammatory reactive thrombocytosis. The differences of these indicators in our three subgroups also provided effective feature support for model classification. As a natural demographic characteristic, age was closely related to the onset tendency of degenerative endplate osteochondritis in elderly individuals. Serum sodium, as a non-specific marker affected by chronic illness and systemic inflammation, also displayed differential distribution among disease groups and supplemented the model’s discriminative information.

SHAP analysis, derived from game theory, can quantify the independent contribution of each clinical feature to individual prediction results and achieve global and local interpretability, which has become a mainstream method to explain the “black box” problem of machine learning models in clinical medical research (Lundberg and Lee, 2017). In this study, SHAP results not only ranked the importance of the 12 screened features but also clarified the positive or negative correlation between each indicator and the predicted probability of each disease, further verifying the clinical rationality of the model decision logic.

As an extended application of the research findings, we encapsulated the established LASSO regression model into a publicly available web-based calculator based on the Shiny platform. This tool only requires the input of 12 easily accessible routine indicators, enabling convenient auxiliary prediction for clinicians at all levels. It has certain potential value for preliminary screening and differential diagnosis in primary medical institutions and grassroots clinical settings. Nevertheless, considering the limitations of single-center data and unbalanced sample size, the clinical popularization and large-scale application of this tool still require further external prospective validation.

Several limitations of this study should be frankly acknowledged. First, this is a single-center retrospective study without multi-center external prospective validation, which limits the generalizability of the model to populations and medical institutions in different regions. Second, the sample size of the PS group is relatively small, and the inter-group sample imbalance leads to only moderate diagnostic efficacy for PS; accordingly, the conclusion of good overall stability of the model cannot be overemphasized. Third, this study only adopted routine serological and clinical indicators, without incorporating imaging features or microbiological examination results; the prediction accuracy still has room for further optimization.

In conclusion, this study constructed an interpretable ternary classification ML model for differentiating STB, PS, and endplate osteochondritis based on readily available clinical laboratory parameters. The model achieved acceptable diagnostic performance for STB and endplate osteochondritis, while the predictive ability for PS remained at a moderate level due to unbalanced sample distribution. We further developed a simple online prediction tool to facilitate clinical auxiliary decision-making. On the premise of fully recognizing the limitations of single-center retrospective design, insufficient PS sample size, and lack of external validation, this study still provides a preliminary exploratory reference for the differential diagnosis of three easily confused spinal diseases. Future work should expand to multi-center samples and conduct external validation to further optimize model performance and confirm its clinical utility.

## Data Availability

The data analyzed in this study is subject to the following licenses/restrictions: Due to the involvement of patients’ personal information and privacy, it is not appropriate to disclose it. Requests to access these datasets should be directed to Tuo Liang, 469693260@qq.com.
